# The Internet and evidence-based decision-making: a needed synergy for efficient knowledge management in health care

**DOI:** 10.2196/jmir.2.suppl2.e2

**Published:** 2000-09-13

**Authors:** Alex Jadad

**Affiliations:** ^1^Health Information UnitMcMaster UniversityCanada

## Abstract

The Internet is introducing new ways for humans to interact with machines and to communicate with each other. In health care the Internet is providing unprecedented opportunities to access information, improve decisions, and enhance communication among decision-makers and the people affected by their decisions.

However, the Internet is also creating many new problems. Seeking information on the Internet is often time-consuming. Internet users, regardless of their role, background or knowledge, can experience confusion and anxiety because of the virtually unlimited amount of information available, information that is often poorly organized and of highly variable quality and relevance.1 The Internet can also lead to conflict among decision-makers if they have access to different and contradictory information. A person's health might even be worsened if inaccurate information found on the Internet were used by decision-makers.

Evidence-based decision-making involves the explicit, conscientious and judicious consideration of the best available evidence in making health care decisions.2 It is supported by a rapidly evolving set of methods and tools but its eventual adoption will depend on whether the barriers it still faces3 can be minimized or eliminated.

In this paper we postulate that if the Internet and evidence-based decision-making are to reach their full potential and contribute to improvements in health care, a powerful and efficient synergy must develop between them.4 The Internet could benefit evidence-based decision-making by giving decision-makers cheap, fast and efficient access to up-to-date, valid and relevant knowledge at the right time, at the right place, in the right amount and in the right format. Conversely, the tools and principles of evidence-based medicine could be used to gain a better understanding of the role of the Internet in health care, helping us to anticipate opportunities and prevent potential problems.

This article briefly describes some of the efforts that are already fostering convergence and synergy between the Internet and evidence-based decision-making, as well as the opportunities available and the challenges to be overcome.

**Full paper published as:**

A. R. Jadad, R. B. Haynes, D. L. Hunt, and G. P. Browman. The Internet and evidence-based decision-making: a needed synergy for efficient knowledge management in health care. CMAJ 162 (3):362-365, 2000. (http://www.cma.ca/cmaj/vol-162/issue-3/0362.htm)

